# The inflammatory potential of the diet in childhood is associated with cardiometabolic risk in adolescence/young adulthood in the ALSPAC birth cohort

**DOI:** 10.1007/s00394-022-02860-9

**Published:** 2022-05-20

**Authors:** Genevieve Buckland, Kate Northstone, Pauline M. Emmett, Caroline M. Taylor

**Affiliations:** 1 Centre for Academic Child Health, Bristol Medical School, University of Bristol, Canynge Hall, 39 Whatley Road, Bristol, BS8 2PS UK; 2 Department of Population Health Sciences, Bristol Medical School, University of Bristol, Bristol, UK

**Keywords:** Cardiometabolic Risk Score, Children and Adolescents, Avon Longitudinal Study of Parents and Children (ALSPAC), Prospective Cohort Study, Dietary Inflammatory Score

## Abstract

**Purpose:**

This study examined the association between a Dietary Inflammatory Score adapted for children (cDIS) and Cardiometabolic Risk (CMR) score in adolescence/early adulthood in the Avon Longitudinal Study of Parents and Children (ALSPAC).

**Methods:**

The cDIS was calculated at 7, 10 and 13 years using diet diary data. Anthropometric and biochemical data at 17 (*N* = 1937) and 24 (*N* = 1957) years were used to calculate CMR scores at each age [mean sex-specific *z*-scores from triacylglycerol, HDL-cholesterol, LDL-cholesterol, mean arterial blood pressure (MAP), homeostatic model assessment of insulin resistance (HOMA-IR) and fat-mass index (FMI)]. Multivariable linear regression models examined associations between cDIS at 7, 10 and 13 years and a continuous CMR *z*-score and individual CMR markers at 17 and 24 years.

**Results:**

In fully adjusted models, a higher cDIS (more pro-inflammatory diet) at 7 years was associated with an increase in CMR z-score at 17 years (*β* 0.19; 95% CI 0.03–0.35 for third versus first cDIS tertile) and at 24 years (*β* 0.28; 95% CI 0.11,0.44 for third versus first cDIS tertile). There was a weak association between a higher cDIS at 10 years and an increase in CMR *z*-score at 17 years (*β* 0.16; 95% CI − 0.003, 0.32 for third versus first cDIS tertile). No other clear associations were evident. FMI, MAP and HOMA-IR were the main CMR factors contributing to these associations.

**Conclusion:**

A more pro-inflammatory diet during childhood was associated with a worse cardiometabolic profile in late adolescence/early adulthood. A childhood diet abundant in nutrients with anti-inflammatory properties could help reduce development of CMR factors.

**Supplementary Information:**

The online version contains supplementary material available at 10.1007/s00394-022-02860-9.

## Introduction

Inflammation plays an important role in the development of cardiometabolic diseases and their risk factors [1–3] and diet may be a key driver of systemic inflammation [4–7]. Extensive research in adults has established that unhealthy dietary patterns such as ‘Western’ diets (generally high in red meat, refined cereals, processed foods and saturated fat, sodium and added sugars) are associated with increased levels of pro-inflammatory biomarkers including C-reactive protein (CRP), E-selectin, tumour necrosis factor-alpha (TNF-α) and interleukin-6 (IL-6) [8, 9] and cardiometabolic risk (CMR) factors [9–11]. In contrast, more healthy dietary patterns such as prudent, health-conscious or Mediterranean diets (generally high in fruit and vegetables, whole-grains, legumes and nuts and healthy oils and sometimes seafood) have been linked with lower levels of similar pro-inflammatory markers [5, 8, 12] and a better CMR profile [11, 13]. Dietary exposures during childhood and adolescents have been associated with inflammatory and cardiometabolic alterations in some but not all studies [14–17] and with markers of subclinical atherosclerosis [18–20]. Therefore, the evidence suggests that the connection between diet quality throughout the life-course and cardiometabolic health is linked to the diet’s ability to exacerbate or ameliorate chronic low-grade systemic inflammation [21, 22].

Research into dietary-associated inflammation and health outcomes has been facilitated by the development of the Dietary Inflammatory Index (DII) [23]. The DII summarises the inflammatory potential of an individual’s overall diet using a continuous scale from anti-inflammatory (negative scores) to pro-inflammatory (positive scores) [23], and has been validated against inflammatory markers [24–26]. The Children’s Dietary Inflammatory Index (C-DII) was subsequently developed for use in paediatric research and uses a similar construct methodology but with a reduced number of dietary parameters (25 compared to 45 items in the DII) [27]. Higher DII and C-DII scores in children have been positively associated with inflammatory biomarkers such as CRP, IL-1, -2 and -6, TNF-α and soluble vascular cell adhesion molecule-1 [17, 27, 28].

Meta-analyses and systematic reviews of observational studies in adults have shown that more pro-inflammatory diets, measured using DII scores, are associated with increased risk of the metabolic syndrome [29], individual cardiometabolic risk factors such as hyperglycaemia and hypertension [30], CVD incidence and mortality [29, 31], and overall mortality [29, 32]. However, the consequence of more pro-inflammatory diets earlier in life on cardiometabolic health is still relatively unknown. A recent (2021) systematic review on the dietary inflammatory potential and cardiometabolic risk and inflammation in children and adolescents only identified four studies (two cross-sectional and two prospective) assessing individual cardiometabolic risk factors including adiposity measures, blood pressure, glucose metabolism, lipid profiles [28]. The only CMR marker in these studies which was consistently related to a DII score was adiposity. However, two cross-sectional studies published after the systematic review reported that a more pro-inflammatory diet in childhood was associated with increased systolic blood pressure (SBP) [33] and markers of dyslipidaemia [34]. Two cohort studies have examined whether the C-DII/DII in childhood was related to a composite metabolic risk score and found opposing results [35, 36]

CMR scores typically incorporate measures of adiposity, lipid profiles, glucose metabolism and blood pressure into a continuous risk score and provide a useful summary of overall cardiometabolic health which can facilitate epidemiological research [37]. To our knowledge, no prospective study in the UK has evaluated how an inflammatory diet during childhood is related to overall cardiometabolic health in early adulthood. Therefore, we investigated whether the inflammatory potential of the diet in children aged 7, 10 and 13 years, measured using a children’s Dietary Inflammatory Score (cDIS), adapted from the C-DII [27], was associated with their CMR score in adolescence and young adulthood (17 and 24 years) in the Avon Longitudinal Study of Parents and Children (ALSPAC).

## Methods

### Cohort description

The study participants were the index children of ALSPAC. ALSPAC is an ongoing British birth cohort established in the 1990s to investigate the determinants of health and disease across the life course [38]. Full details of the study have been reported previously [39–42] and are also available on the ALSPAC website (www.alspac.bris.ac.uk). In summary, 14,541 eligible pregnant women from the South-West of England were initially enrolled into the study in 1991–1992, resulting in 13,988 children alive at 1 year. Two subsequent recruitment phases [42] in 1999 (child mean age: 7.5 years) and in 1999–2012 (child mean age: 17.8 years) provided a final sample of 14,869 eligible children (after excluding participants who withdrew consent and triplet and quadruplet pregnancies for reasons of confidentiality). During periodic follow-ups, extensive data have been collected from the parents and their children, primarily using questionnaires, medical records and face to face visits. Study data were collected and managed using Research Electronic Data Capture (REDCap) electronic data capture tools hosted at the University of Bristol [43]. REDCap is a secure, web-based software platform designed to support data capture for research studies. The study website contains details of all the data that are available through a fully searchable data dictionary and variable search tool (http://www.bristol.ac.uk/alspac/researchers/our-data/). Ethical approval for the study was obtained from the ALSPAC Ethics and Law Committee and the Local Research Ethics Committee (http://www.bristol.ac.uk/alspac/researchers/research-ethics/) and conformed to the Declaration of Helsinki. Consent for biological samples was collected in accordance with the Human Tissue Act (2004).

### Dietary assessment

**Fig. 1:**
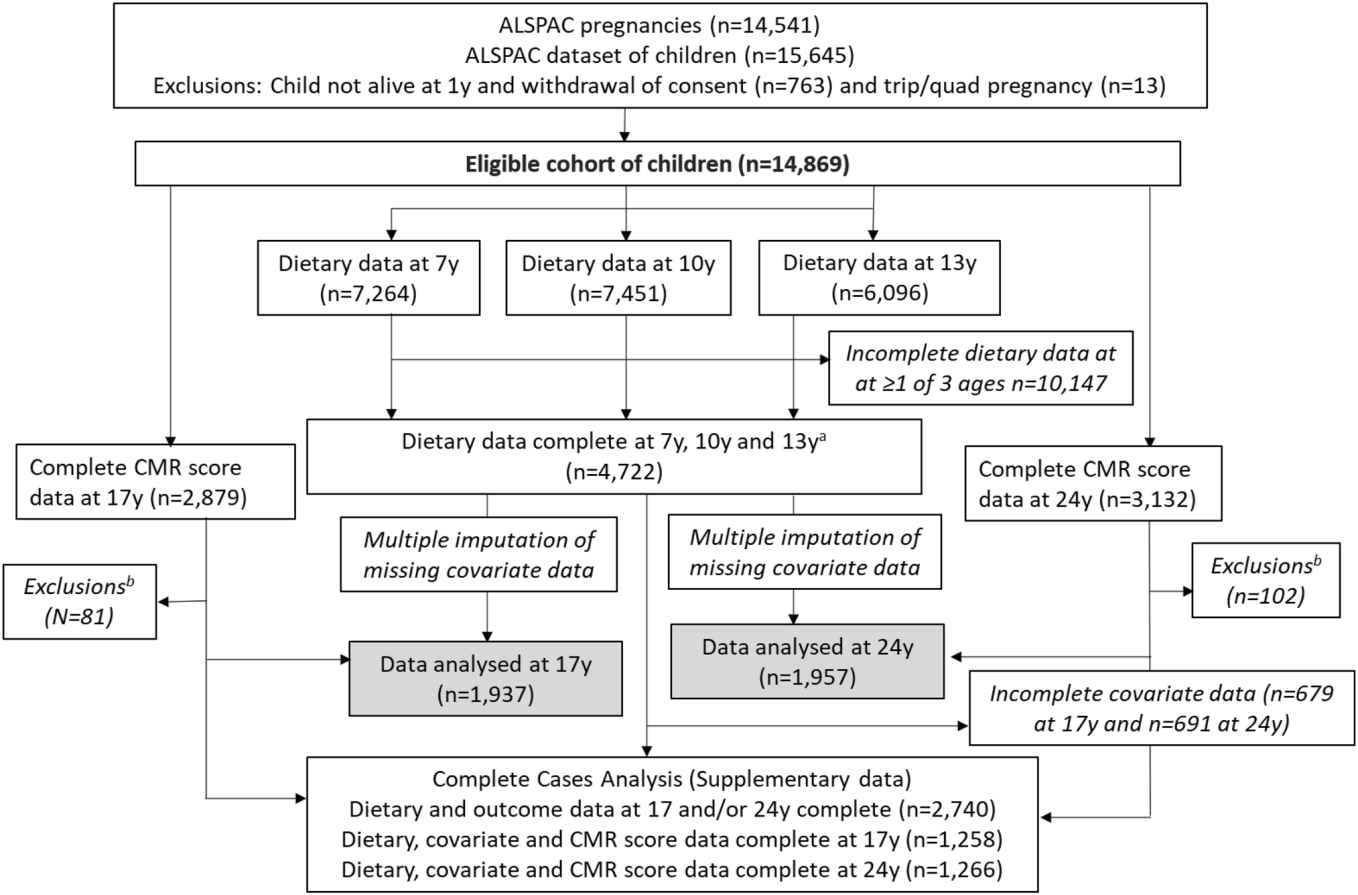
Study Flow Diagram for participant data from the Avon Longitudinal Study of Parents and Children (ALSPAC). The present study uses data from participants with complete dietary data at 7, 10 and 13 years and complete data on the cardiometabolic parameters to derive the CMR score at 17 years and 24 years and uses multiple imputation for missing data in covariates. ^a^Complete dietary data refers to at least one diet diary recorded for a child at all three ages (7, 10 and 13 years). Three complete days of diet diary data were available for 86.5%, 83.6% and 78.4% of children at 7, 10 and 13 years, respectively. ^b^Exclusions were participants with diagnosed diabetes, on insulin treatment or fasting glucose level ≥ 7 mmol/L and subjects with extreme outliers, defined as more than 4sd from the mean, on any of the six CMR score components

Children were invited to attend research clinics at the ages of 7, 10 and 13 [mean age at attendance: 7.5 (sd = 0.3), 10.6 (sd = 0.2) and 13.8 (sd = 0.2)] years. Prior to each of these clinics a 3-day diet diary was sent to them for completion, recording all food and drink consumed over two weekdays and one weekend day. This was completed by the caregiver when the child was 7 years and by the children with assistance from an adult when the children were 10 and 13 years. During the clinic visits at 10 and 13 years a nutritionist checked the diaries for completeness or discrepancies and clarified portion sizes. The completed diaries were coded and linked to food composition tables using DIDO (Diet In Data Out). McCance and Widdowson’s British food composition data were used to calculate nutrient intakes [44]. Validity of dietary reporting was calculated using an individualised method based on the ratio of energy intake to estimated energy requirement and its 95% confidence interval [45]. Further details on the ALSPAC dietary assessment methods have been published previously [46]. Data from dietary diaries were available for 7264 children at 7 years, for 7451 at 10 years and for 6096 at 13 years. 4,722 had complete dietary data all three ages (Fig. 1).

### The children’s dietary inflammatory score (cDIS)

The inflammatory potential of the diet was assessed using the cDIS, which was adapted from the C-DII [27] and DII [23]. The C-DII is an index created by assigning inflammatory weights to 25 food components (whole foods, macro- and micro-nutrients, and other dietary constituents) in line with their proinflammatory or anti-inflammatory properties. Each component’s weighting was calculated according to their associations with six key inflammatory markers (CRP, TNF-α, IL-1b, IL-4, IL-6, IL-10) and took into account the strength of evidence from the studies and numbers of articles reviewed [23].

The cDIS included 24 of the 25 components included in the C-DII; we did not include alcohol in the cDIS due to the age groups studied. The 24 dietary components used to construct the cDIS were: energy, carbohydrate, protein, total fat, saturated fat, monounsaturated fat, polyunsaturated fat, dietary cholesterol, fibre, vitamin A, vitamin B6, vitamin B12, vitamin C, vitamin D, vitamin E, folic acid, beta-carotene, thiamine, riboflavin, niacin, iron, magnesium, zinc and selenium. Firstly, the daily intake of each dietary component was expressed as a function of energy density by dividing each participant’s intake by their total daily energy intake and multiplying by 1000 kcal (4.2 MJ). Secondly, each dietary component was standardized using *z*-scores by subtracting the mean of our study population from the participant’s intake and dividing the result by the standard deviation of intake (while the C-DII standardizes the individual dietary components of the score using a composite children’s dietary database [27]). Thirdly, to minimize right skewing the *z*-scores were converted into cumulative proportions (with values ranging from 0 to 1) and then centred around zero by doubling each percentile and subtracting 1 [27]. Fourthly, to calculate the inflammatory score for each dietary component each centred *z*-score was multiplied by its corresponding positive or negative inflammatory weight [23]. Finally, the overall cDIS for each participant was calculated by summing together the inflammatory scores from each of the 24 dietary components. The cDIS is a score without units and expresses an individual’s diet relative to the other participants, at a point along on a continuous scale ranging from below to above zero. A lower cDIS score (negative values) indicates a more anti-inflammatory diet while a higher score (positive score) reflects a more pro-inflammatory diet.

### Cardiometabolic risk factors

CMR factors were assessed using measurements and blood samples collected by study nurses and clinic staff using standardised procedures, when the participants attended study clinics at 17.7 years (sd 0.3) and 24.5 years (sd 0.8). Blood pressure was measured in a seated position and resting state, using an Omron 705 IT and Omron M6 oscillometric recorder (Omron Electronic Components Europe BV) at the 17-year and 24-year clinic, respectively. Systolic blood pressure (SBP) and diastolic blood pressure (DBP) were measured twice on the right arm, using the appropriate cuff size for the upper arm circumference, and the mean of each was recorded. Mean arterial blood pressure (MAP) was then calculated using the formula: 1/3(SBP) + 2/3(DBP) [47]. Blood samples were taken using standard procedures and in a fasting state; participants were asked to fast overnight or at least 6–8 h prior to the clinic visit. The samples were immediately centrifuged and frozen at − 80 °C. The samples collected were assayed 3–9 months later, with no previous freeze–thawing cycles. Plasma lipids (total cholesterol, triacylglycerol, low-density lipoprotein cholesterol (LDL-c) and high-density lipoprotein cholesterol (HDL-c)) were analysed according to the standard Lipid Research Clinics Protocol using enzymatic reagents for lipid determination. Glucose and insulin were used to calculate the homeostatic model assessment of insulin resistance (HOMA-IR) using the following formula: (fasting plasma glucose (mg/dl) × fasting plasma insulin (mU/L))/405 [48]. At each age, participant height was measured to the nearest 0.1 cm using a Harpenden stadiometer (Holtain Ltd, Crymych, Pembs, UK) and weight using the Tanita Body Fat Analyser weighing scale (Tanita, West Drayton, Middlesex, UK). Waist circumference was measured to the nearest millimetre using Seca 201 body tension tape. Height and circumference were measured to the nearest millimetre, while weight was measured to the nearest 0.1 kg. Body mass index (BMI) was calculated as body mass (kg)/height (m)^2^. Fat mass (kg) was assessed at 17 and 24 years using a Lunar Prodigy Dual Emission X-ray Absorptiometry (DXA) scanner (GE Medical Systems, Madison, Wisconsin, USA) and fat mass index (FMI) was calculated as fat mass (kg)/height (m)^2^.

### Cardiometabolic risk score

A composite standardized continuous CMR score was calculated for each participant at 17 and 24 years [37]. The CMR score included six cardiometabolic markers: FMI, HDL-c, LDL-c, triacylglycerol, MAP and HOMA-IR. Complete data on all markers were available for *n* = 2879 at 17 years and *n* = 3132 at 24 years. Participants with diagnosed diabetes (*n* = 17) or on insulin treatment (*n* = 15) and participants with fasting glucose concentration ≥ 7 mmol/L but who had not reported diabetes (*n* = 4 at 17 years and *n* = 14 at 24 years) were excluded from the analysis due to potential problems of using HOMA-IR to assess insulin sensitivity in diabetic subjects [49]. In addition, participants with extreme/implausible values (defined as more than 4 sd from the mean) on any of the six CMR score components were excluded (*n* = 42 at 17 years and *n* = 52 at 24 years). CMR scores were calculated for participants who had complete dietary data at 7, 10 and 13 years and complete outcome data at 17 years (*n* = 1937) and 24 years (*n* = 1957). Each CMR component was log-transformed to normalize the data (due to right skewing for the majority of the biomarkers) and then sex-specific *z*-scores were calculated to standardize the units: (*z*-score(component1) = [individual’s value minus sex-specific sample mean]/sex-specific sample SD). HDL-c was multiplied by − 1, to align the direction of values for increased risk with the other components. The *z*-scores from the six CMR components were summed and divided by the square root of six (to preserve the *z*-score distribution) [37], to give a final CMR score for each participant at 17 years and at 24 years.

### Covariates

Data on sex, birth weight and gestational age at birth were collected by ALSPAC staff at delivery, from medical records or from birth notifications. Maternal age at delivery was derived from maternal date of birth and child’s date of birth. Exact age (months) of study participants was recorded at all clinics. Maternal data were collected by self-completion postal questionnaires during pregnancy. Maternal education was reported as the highest completed out of Certificate of Secondary Education (CSE), vocational training, O-level/General Certificate of Secondary Education (qualifications obtained at 16 years of age), A-levels (qualification obtained at 18 years), University degree or higher. Maternal and paternal social class were derived using the 1991 Office of Population Censuses and Surveys (OPCS) occupation-based classification, based on the current or last job at 32 weeks of gestation. This resulted in standardised UK social class classifications: class I (highest), II, III–non-manual, III–manual, IV and V [50]. Maternal and paternal social class were combined to give highest family social class. The participant’s physical activity was assessed at 13 years and 15 years of age using an Actigraph AM7164 2.2 accelerometer (Actigraph LLC, Fort Walton Beach, FL, USA), which was worn around the waist, at the right hip, for 7 consecutive days [51]. A valid day was defined as providing data for at least 10 h and participants were only included in the analyses if they provided at least 3 valid days of recording. Moderate-to-vigorous physical activity (MVPA) was calculated using the mean minutes per day in which there were > 3600 accelerometer counts per minute [51]. Validity of dietary reporting was categorised into under-reporting, valid reporting and over-reporting. Puberty timing was estimated using peak height velocity, previously calculated in ALSPAC [52].

### Statistical analysis

Of the baseline cohort of 14,869 participants alive at one year, 4722 had dietary data available at all three ages (7, 10 and 13 years) and 2,740 had complete dietary data and outcome data at 17 years and/or 24 years (Fig. 1). The cDIS was calculated for these 2740 participants. Baseline characteristics for the 2740 participants and those with data on the components of the CMR score at 17 years (1937) and 24 years (1957) were compared across tertiles of the cDIS categories at the three ages using proportions for categorical variables and means (sd) or medians (inter-quartile ranges (IQR)) for normal and non-parametric continuous variables, respectively. Chi-squared tests were used to assess differences between categorical variables and Kruskal–Wallis tests for continuous variables. The same statistical tests were used to compare participants with complete dietary data at all three ages and outcome data at 17 and/or 24 years (*n* = 2740) to participants with missing dietary data at one or more time points and incomplete outcome data (*n* = 3356 to 11,325 depending on missingness of covariates). The correlation between the continuous CMR score at 17 and 24 years was assessed in the 1154 participants with data on both outcomes. The correlation between the continuous cDIS score between 7, 10 and 13 years was measured using partial Pearson correlation coefficients adjusted for validity of dietary reporting. In line with previous research on correlations of dietary patterns [53], a coefficient of < 0.30 was the cut-off point applied to indicate low correlation, between 0.30 and 0.59 was considered moderate correlation and ≥ 0.60 as high correlation.

Several factors were considered as potential confounders of the association between the cDIS and CMR score: sex, age of child at each dietary assessment and at each outcome, child’s birth weight, gestational age at birth, maternal age at delivery, parity, maternal pre-pregnancy BMI, marital status, mothers’ highest education level, family highest social class, puberty timing, MVPA, dietary misreporting, number of days dietary diary collected, and total energy intake. The association between these potential confounders and the exposure (cDIS at each age) and the outcome (composite CMR score at each age) was evaluated in bivariate analyses (data not shown). For categorical variables chi-square tests were conducted and Kruskal–Wallis tests were used for continuous variables. Only covariates which were related to both the exposure and outcome (*p* < 0.10) were included in the regression models. The variance inflation factors for the covariates in all the different models were all ≤ 5, so the assumption of no multicollinearity was supported.

The association between the exposure (cDIS at 7, 10 and 13 years) and outcome (CMR score at 17 years and 24 years) was assessed using minimally adjusted (sex and validity of dietary reporting) and fully adjusted multivariable linear regression models (additionally adjusted for mother’s highest education level, family highest social class, MVPA at 11 years and 13 years). The cDIS was assessed as categorical (tertiles; first tertile as reference) and continuous (per 1-unit increment) variables. The CMR score was assessed as a continuous variable (per unit increase). The standardised regression coefficients (beta-coefficients) represent the estimated mean change in CMR *z*-score associated with a 1 unit increase in cDIS. Results were presented for both sexes together, as there was no evidence that the relation between the cDIS and CMR score was modified by sex in any of the models (lrtest comparing models with and without an interaction term between the cDIS and sex resulted in all *p* values > 0.10). The likelihood ratio test was used to assess the shape of the association by comparing the model using a categorical cDIS variable to the respective model using a continuous cDIS variable. In complementary analyses we further adjusted for child’s BMI at dietary data collection.

Multivariable linear regression models were also used to assess the association between the cDIS (per 1-unit increment) and each individual log-transformed z-score of CMR score components (FMI, HDL-c, LDL-c, triacylglycerol, MAP and HOMA-IR) and additional CMR factors (BMI, waist circumference, SPB, DBP, total cholesterol, insulin and glucose). A combined cDIS tracking variable was also constructed by taking into account each participant’s cDIS tertile group at 7, 10 and 13 years, resulting in four mutually exclusive categories: low: first tertile at least twice, mixed: different tertiles at all ages, medium: second tertile at least twice, high: third tertile at least twice. All statistical analyses were performed using Stata version 15.1 (Stata Corporation, College Station, Texas, USA).

### Missing data and multiple imputation

Missing covariate data were imputed using multiple imputation. Chained equations (ICE command) in Stata generated twenty stacked datasets which were used in the final regression analyses, with standard combination rules for multiple imputations [54]. The variables included in the imputation models were all of those included in the final regression models and additional auxiliary variables which also strongly predicted missingness in the covariates (Family Adversity Index [55], maternal pre-pregnancy BMI, child’s BMI and total energy intake at each time of dietary data collection). Thus, the basic assumption underlying multiple imputation that data were ‘missing at random’ [54] was supported. Separate imputed datasets were created for the participants with complete dietary and CMR data at 17 years (*n* = 1957) and at 24 years (*n* = 1957). The results in the main article are derived from the regression analysis using the imputed datasets, while results from complete-case analyses for CMR at 17 years (*n* = 1258) and CMR at 24 years (*n* = 1266) are presented in the supplementary material.

## Results

The final study sample included 1,937 (50% female) participants with cDIS and CMR score data at 17 years and 1,957 (57% female) participants with cDIS and CMR score data at 24 years (Fig. 1). Participants with complete dietary and CMR data (*n* = 2740) were more likely to be female, had a lower BMI at 10 years, mothers with a higher education level, a higher family social class and in general had a better cardiometabolic profile at 17 and 24 years, when compared to participants with missing dietary and CMR data (Supplementary Table 1). A comparison of the imputed and observed data (Supplementary Table 2) showed that the two datasets were very similar in terms of distribution between subgroups of each covariate.

At 7 years the cDIS had a mean of 0.20 (sd 1.5), median of 0.25 and ranged from − 5.1 (maximum anti-inflammatory diet) to 4.3 (maximum pro-inflammatory diet). At 10 years the cDIS had a mean of 0.20 (sd 1.5), median of 0.23 and ranged from − 4.6 to 4.5. At 13 years the cDIS had a mean of 0.23 (sd 1.6), median of 0.35 and ranged from − 4.9 to 4.5. The median intake and interquartile range for each of the 24 dietary components included within the cDIS at 7, 10 and 13 years is detailed in Supplementary Table 3. The CMR score at 17 years had a mean of 0 (sd 1.45) and ranged from − 4.19 to 5.27, and the CMR at 24 years at 24 years had a mean of 0 (sd 1.55) and ranged from − 4.50 to 5.11.

The baseline characteristics of the 2740 participants according to tertiles of cDIS at 7, 10 and 13 years are shown in Table 1. Participants in the lower cDIS tertile (more anti-inflammatory diet) at all three ages had a lower total energy intake, were more likely to have mothers with a lower BMI and higher level of education and had a higher family social class, compared to those in the upper cDIS tertile (more pro-inflammatory diet). Participants with a lower cDIS (in the first compared to third tertile) at 13 years had a lower MAP but a higher FMI and level of triacylglycerol at 17 years (Table 2). Participants with a lower cDIS at 10 years had a lower HOMA-IR score at 17 years. FMI, MAP and HOMA-IR score at 24 years were higher in participants who had higher cDIS scores (more pro-inflammatory diet) at 7 years.

**Table 1:** Characteristics of the study sample with complete dietary and outcome data, according to tertile categories of the children’s Dietary Inflammatory Score (cDIS) at 7, 10 and 13 years

Characteristics of the ALSPAC index children	*N*	cDIS at 7 years			cDIS at 10 years			cDIS at 13 years		*p* value
		T1	T3	*p* value	T1	T3	*p* value	T1	T3	
		Mean (SD)			Mean (SD)			Mean (SD)		
cDIS	2740	− 1.5 (0.8)	1.8 (0.7)	0.001	− 1.5 (0.8)	1.9 (0.7)	0.001	− 1.5 (0.9)	1.9 (0.7)	0.001
Age^a^, years	2740	7.5 (0.3)	7.5 (0.3)	0.385	10.6 (0.2)	10.6 (0.2)	0.162	13.8 (0.2)	13.8 (0.2)	0.070
BMI^b^, kg/m^2^	2450	16.0 (1.9)	16.2 (2.0)	0.165	18.0 (2.8)	18.0 (3.0)	0.850	20.5 (3.3)	20.1 (3.1)	< 0.001
Total Energy, kJ/day	2740	6896 (1230)	7553 (1323)	< 0.001	7441 (1402)	8321 (1639)	< 0.001	7566 (1838)	8944 (2213)	< 0.001

Characteristics of the ALSPAC index children	*N*	cDIS at 7 years			cDIS at 10 years			cDIS at 13 years		*p* value
		T1	T3	*p* value	T1	T3	*p* value	T1	T3	
		%			%			%		
Sex, female	2740	53.0	53.9	0.733	53.1	52.9	0.958	59.4	48.5	< 0.001
Puberty timing, early	2662	51.3	52.5	0.663	52.7	50.9	0.576	52.1	51.7	0.708
Maternal age at delivery, ≥ 30 years	2632	53.6	48.0	0.058	53.8	48.2	0.065	54.1	48.6	0.065
Maternal pre-pregnancy BMI, ≥ 25 kg/m^2^	2450	13.5	21.3	< 0.001	15.2	19.8	0.042	17.2	18.0	0.777
Maternal education, up to A-level/Degree	2595	56.4	47.9	0.002	58.0	47.4	< 0.001	57.0	46.4	< 0.001
Highest household social class up to Grade I/II	2532	39.6	29.4	< 0.001	38.9	28.5	< 0.001	40.5	29.7	< 0.001
Moderate-to-vigorous physical activity at 11 years ≥ 20 min/day	2336	49.9	47.6	0.660	49.7	45.8	0.116	47.0	50.5	0.375

*cDIS* Children's Dietary Inflammatory Score (energy-adjusted), *T* Tertile

^a^Age refers to age at each point of dietary data collection (7, 10, 13 years). ^b^BMI corresponds to measurements taken at each time point of dietary data collection

**Table 2:** Cardiometabolic Risk Factors at 17 years and 24 years in the sample with complete dietary and outcome data, according to tertile categories of the children’s Dietary Inflammatory Score (cDIS) at 7, 10 and 13 years

Cardiometabolic risk factors	*N*	cDIS at 7 years (median (IQR))			cDIS at 10 years (median (IQR))			cDIS at 13 years (median (IQR))		*p* value
		T1	T3	*p* value	T1	T3	*p* value	T1	T3	
*17 years*										
Fat Mass Index, kg/m^2^	1937	5.1 (3.0–7.7)	5.6 (3.1–7.9)	0.465	5.3 (3.1–7.5)	5.4 (3.1–7.7)	0.752	5.6 (3.7–7.8)	5.1 (2.8–7.7)	0.025
HDL-cholesterol, mmol/L	1937	1.2 (1.1–1.5)	1.2 (1.1–1.5)	0.395	1.2 (1.1–1.4)	1.2 (1.1–1.5)	0.101	1.2 (1.0–1.5)	1.2 (1.0–1.4)	0.666
LDL-cholesterol, mmol/L	1937	2.0 (1.7–2.5)	2.1 (1.7–2.5)	0.106	2.1 (1.7–2.5)	2.0 (1.7–2.5)	0.941	2.1 (1.7–2.5)	2.0 (1.7–2.5)	0.677
Triacylglycerol, mmol/L	1937	0.8 (0.6–1.0)	0.7 (0.6–1.0)	0.464	0.7 (0.6–0.9)	0.7 (0.6–1.0)	0.485	0.8 (0.6–1.0)	0.7 (0.6–1.0)	0.030
MAP, mmHg^a^	1937	80.5 (77.1–84.8)	81.3 (77.6–85.1)	0.099	80.6 (76.9–84.5)	81.2 (77.8–84.9)	0.080	80.7 (76.9–84.2)	81.1 (77.8–85.6)	0.018
HOMA-IR^b^	1937	1.5 (1.0–2.0)	1.5 (1.1–2.1)	0.064	1.4 (1.0–1.9)	1.5 (1.1–2.1)	0.009	1.4 (1.1–2.0)	1.5 (1.1–2.1)	0.395
*24 years*										
Fat Mass Index, kg/m^2^	1957	6.7 (5.0–8.8)	7.0 (5.4–9.6)	0.022	6.7(5.2–8.8)	6.8 (5.2–9.0)	0.550	7.0 (5.4–9.3)	6.7 (5.1–9.1)	0.287
HDL-cholesterol, mmol/L	1957	1.5 (1.3–1.8)	1.5 (1.3–1.8)	0.087	1.5 (1.3–1.8)	2.3 (1.9–2.9)	0.214	1.5 (1.3–1.8)	1.5 (1.2–1.8)	0.108
LDL-cholesterol, mmol/L	1957	2.3 (1.8–2.8)	2.4 (2.0–2.9)	0.264	2.3 (1.9–2.9)	2.3 (1.9–2.9)	0.890	2.3 (1.9–2.9)	2.4 (1.9–2.9)	0.563
Triacylglycerol, mmol/L	1957	0.8 (0.6–1.1)	0.8 (0.6–1.1)	0.106	0.8 (0.6–1.1)	0.8 (0.6–1.1)	0.921	0.8 (0.6–1.1)	0.8 (0.7–1.1)	0.104
MAP, mmHg^a^	1957	81.2 (76.8–87.1)	83.1 (78.6–88.0)	0.054	82.4 (77.2–87.7)	82.6 (78.1–88.1)	0.193	81.8 (76.9–87.3)	83.1 (78.3–88.3)	0.146
HOMA-IR^b^	1957	1.6 (1.1–2.4)	1.8 (1.2–2.5)	0.017	1.7 (1.2–2.4)	1.7 (1.2–2.5)	0.361	1.7 (1.2–2.4)	1.7 (1.2–2.5)	0.516

*cDIS* Children's Dietary Inflammatory Score, *IQR* Inter-quartile range (25th–75th percentile), *T* Tertile, *HDL* High-density lipoprotein cholesterol, *LDL* Low-density lipoprotein cholesterol, *MAP* Mean arterial blood pressure, *HOMA-IR* Homeostatic Model Assessment of Insulin Resistance

^a^MAP calculated as (2 × (diastolic blood pressure) + (systolic blood pressure)/3. ^b^HOMA-IR calculated as (fasting plasma glucose(mg/dl) × fasting plasma insulin(mU/L))/405

The correlation coefficient for continuous cDIS between 7 and 10 years was 0.34 (95% CI: 0.30, 0.37), between 7 and 13 years it was 0.25 (95% CI: 0.22, 0.29) and between 10 and 13 years it was 0.30 (95% CI 0.27, 0.34), indicating moderate correlation of the cDIS from 7 to 10 year and from 10 to 13 years and low correlation from 7 to 13 years. The correlation coefficient for the continuous CMR score at 17 and 24 years was 0.56 (95% CI 0.52, 0.60), indicating a moderate-to-high correlation of CMR score between these ages.

The crude and adjusted associations between the cDIS (tertiles and per unit increment) at 7, 10 and 13 years and CMR score at 17 years and 24 years are shown in Tables 3 and 4, respectively. In fully adjusted models, a higher cDIS at 7 was associated with an increase in CMR score at 17 years (*β* 0.19; 95% CI 0.03, 0.35 for third compared to first cDIS). There was a weak association between the cDIS at 10 years and CMR score at 17 years (*β* 0.16; 95% CI − 0.003, 0.32 for third compared to first cDIS). There was no evidence of an association between the cDIS at 13 years and CMR score at 17 years. There was a similar pattern of results for the CMR score at 24 years; a higher cDIS at 7 was related to increased CMR score, there was a weak association between the cDIS at 10 years continuous CMR score and there was no association with the cDIS at 13 years. The strongest association was observed between the cDIS at 7 years and CMR at 24 years (*β* 0.28; 95% CI 0.11, 0.44 for third compared to first cDIS tertile in fully adjusted models). The likelihood ratio test comparing the categorical cDIS with the continuous cDIS, in all the combination of exposure and outcome models supported the assumption that the associations were linear (*p* > 0.05). The combined cDIS tracking variable (accounting for combinations of cDIS tertile categories at 7, 10 and 13 years) was also associated with the CMR score at 17 years (*p*-trend = 0.042) and 24 years (*p*-trend = 0.015), although the evidence was not as strong for CMR at 17 years as only the ‘high’ category, where individuals were in the top cDIS tertile at least twice between 7 and 13 years, was associated with the CMR score.

**Table 3:** Multivariable linear regression models for the relationship between the children’s Dietary Inflammatory Score (cDIS) at 7, 10 and 13 years and cardiometabolic risk score at 17 years, using imputed datasets in the ALSPAC cohort (*n* = 1937)

Children's Dietary Inflammatory Score (cDIS)^a^	Cardiometabolic Risk (CMR) score at 17 years						
	*N*	Crude		Minimally adjusted^b^		Fully adjusted^c^	
		*β* (95% CI )	*p* value	*β* (95% CI )	*p* value	*β* (95% CI )	*p* value
*cDIS at 7 years*							
Tertile 1	645	Reference		Reference		Reference	
Tertile 2	645	− 0.48 (− 0.21, 0.11)	0.555	− 0.06 (− 0.21, 0.10)	0.487	− 0.04 (− 0.20, 0.11)	0.593
Tertile 3	647	− 0.16 (− 0.001, 0.31)	0.052	0.18 (0.03, 0.34)	0.023	0.19 (0.03, 0.35)	0.022
Continuous^d^	1937	0.05 (0.01, 0.09)	0.021	0.06 (0.02, 0.10)	0.006	0.06 (0.02, 0.11)	0.006
*cDIS at 10 years*							
Tertile 1	640	Reference		Reference		Reference	
Tertile 2	645	0.05 (− 0.11, 0.21)	0.517	0.09 (− 0.07, 0.25)	0.252	0.07 (− 0.09, 0.23)	0.370
Tertile 3	652	0.12 (− 0.03, 0.28)	0.122	0.21 (0.05, 0.37)	0.011	0.16 (− 0.003, 0.32)	0.054
Continuous^d^	1937	0.05 (0.004, 0.09)	0.032	0.07 (0.03, 0.11)	0.002	0.06 (0.01, 0.10)	0.012
*cDIS at 13 years*							
Tertile 1	633	Reference		Reference		Reference	
Tertile 2	660	− 0.06 (− 0.22, 0.10)	0.466	0.00 (− 0.16, 0.16)	0.986	− 0.02 (− 0.18, 0.14)	0.803
Tertile 3	644	0.03 (− 0.13, 0.19)	0.715	0.13 (− 0.04, 0.29)	0.131	0.09 (− 0.07, 0.26)	0.280
Continuous^d^	1937	0.005 (− 0.04, 0.05)	0.831	0.03 (− 0.01, 0.08)	0.125	0.02 (− 0.02, 0.07)	0.285
*cDIS tracking 7–10–13 years*^e^							
cDIS low	566	Reference		Reference		Reference	
cDIS mixed	296	0.01 (− 0.20, 0.21)	0.951	0.04 (− 0.17, 0.24)	0.721	0.00 (− 0.20, 0.21)	0.980
cDIS medium	517	− 0.07 (− 0.24, 0.10)	0.423	− 0.02 (− 0.19, 0.16)	0.851	− 0.05 (− 0.22, 0.12)	0.566
cDIS high	558	0.14 (− 0.03, 0.31)	0.095	0.26 (0.09, 0.43)	0.003	0.21 (0.04, 0.38)	0.017

^a^cDIS: energy adjusted children's Dietary Inflammatory score. ^b^Minimally adjusted: Multivariable regression model adjusted for sex and dietary misreporting. ^c^Fully adjusted: Multivariable regression model adjusted for sex, dietary misreporting, maternal highest education level, family highest social class and physical activity level at 11 years (for analysis of cDIS at 7 and 10 years) and physical activity at 13 years (for analysis of cDIS at 13 years). ^d^Estimated mean change in CMR *z*-score associated with a 1 unit increase in cDIS. ^e^cDIS tracking; low indicates first tertile of cDIS at least twice from 7–10–13 years, mixed indicates different tertiles of cDIS at 7–10–13 years, medium indicates second tertile of cDIS at least twice from 7–10–13 years, high indicates high tertile of cDIS at least twice from 7–10–13 years

**Table 4:** Multivariable linear regression models for the relationship between the children’s Dietary Inflammatory Score (cDIS) at 7, 10 and 13 years and cardiometabolic risk score at 24 years, using imputed datasets in the ALSPAC cohort (*n* = 1957)

children's Dietary Inflammatory Score (cDIS)^a^	Cardiometabolic Risk (CMR) score at 24 years						
	*N*	Crude		Minimally adjusted^b^		Fully adjusted^c^	
		*β* (95% CI )	*p* value	*β* (95% CI )	*p* value	*β* (95% CI )	*p* value
*cDIS at 7 years*							
Tertile 1	660	Reference		Reference		Reference	
Tertile 2	657	0.25 (0.08, 0.41)	0.004	0.28 (0.12, 0.45)	0.001	0.26 (0.09, 0.42)	0.002
Tertile 3	640	0.27 (0.10, 0.43)	0.002	0.33 (0.17, 0.50)	< 0.001	0.28 (0.11, 0.44)	0.001
Continuous^d^	1957	0.08 (0.03, 0.12)	0.001	0.10 (0.06, 0.15)	< 0.001	0.08 (0.04, 0.13)	< 0.001
*cDIS at 10 years*							
Tertile 1	657	Reference		Reference		Reference	
Tertile 2	648	0.08 (− 0.09, 0.24)	0.376	0.11 (− 0.05, 0.28)	0.182	0.08 (− 0.08, 0.25)	0.323
Tertile 3	652	0.12 (− 0.05, 0.28)	0.177	0.18 (0.01, 0.35)	0.034	0.12 (− 0.05, 0.29)	0.152
Continuous^d^	1957	0.04 (− 0.004, 0.09)	0.073	0.06 (0.02, 0.11)	0.007	0.04 (− 0.001, 0.09)	0.054
*cDIS at 13 years*							
Tertile 1	656	Reference		Reference		Reference	
Tertile 2	656	0.08 (− 0.08, 0.25)	0.332	0.13 (− 0.04, 0.29)	0.144	0.08 (− 0.08, 0.25)	0.322
Tertile 3	645	0.09 (− 0.07, 0.26)	0.276	0.18 (0.01, 0.35)	0.042	0.11 (− 0.06, 0.28)	0.209
Continuous^d^	1957	0.03 (− 0.01, 0.07)	0.167	0.06 (0.01, 0.11)	0.010	0.04 (− 0.005, 0.08)	0.079
*cDIS tracking 7–10–13 years*^e^							
cDIS low	593	Reference		Reference		Reference	
cDIS mixed	285	0.35 (0.13, 0.57)	0.002	0.36 (0.14, 0.57)	0.001	0.28 (0.06, 0.50)	0.012
cDIS medium	521	0.22 (0.04, 0.40)	0.017	0.27 (0.09, 0.45)	0.003	0.24 (0.06, 0.41)	0.010
cDIS high	558	0.20 (0.03, 0.38)	0.025	0.30 (0.12, 0.48)	0.001	0.22 (0.04, 0.41)	0.015

^a^cDIS: energy adjusted children's Dietary Inflammatory score. ^b^Minimally Adjusted: Multivariable regression model adjusted for sex and dietary misreporting. ^c^Fully Adjusted: Multivariable regression model adjusted for sex, dietary misreporting, maternal highest education level, family highest social class and physical activity level at 11 years (for analysis of cDIS at 7 and 10 years) and physical activity at 13 years (for analysis of cDIS at 13 years). ^d^Estimated mean change in CMR *z*-score associated with a 1 unit increase in cDIS. ^e^cDIS tracking; low indicates first tertile of cIDS at least twice from 7–10–13 years, mixed indicates different tertiles of cDIS at 7–10–13 years, medium indicates second tertile of cDIS at least twice from 7–10–13 years, high indicates high tertile of cDIS at least twice from 7–10–13 years

The results from a complete-case analysis (Supplementary Tables 4 and 5) were very similar, although for some associations the effect sizes were slightly larger than in the imputed analysis. In a sensitivity analysis, participants without three days of diet diaries were excluded from the complete-case analysis (13.5%, 16.4% and 21.6% of the 2740 children at 7, 10 and 13 years had 1 or 2 days of diet diary, respectively), which resulted in minimal changes (Supplementary Table 6). Further sensitivity analyses, additionally adjusting for child’s BMI at time of dietary data collection also resulted in only minor differences in the effect estimates (Supplementary Table 6).

The adjusted associations between the cDIS and individual CMR factors (anthropometrics, blood lipids, blood pressure and glucose metabolism) at 17 years and 24 years (all per 1-unit increment of z-scores) are detailed in Tables 5 and 6, respectively. A higher cDIS at 7, 10 and 13 years was positively associated with an increase in several CMR factors at 17 years; BMI, FMI, MAP (largely due to an increase in DBP at 17 years and both SBP and DBP at 24 years) and HOMA-IR (largely due to an increase in insulin). The effect sizes were all relatively small (beta coefficients ranging from 0.02 to 0.05 for findings with evidence of an association). For individual CMR factors at 24 years, the anthropometric measures (BMI, FMI and waist circumference) were generally positively associated with the cDIS at 7, 10 and 13 years. A greater cDIS at 7 years was related to an increase in all the blood pressure measurements (DPB, SPB and MAP) and glucose metabolism (insulin and HOMA-IR score) at 24 years. In contrast the cDIS at 10 and 13 years was not associated with individual CMR factors at 24 years, except for the anthropometric measures previously mentioned. The effect sizes were all relatively small (betas ranging from 0.03 to 0.16 for findings with evidence of an association). The complete-case analysis showed similar pattern of associations with the individual CMR factors (Supplementary Tables 7 and 8).

**Table 5:** Association between the children’s Dietary Inflammatory Score (cDIS) at 7, 10 and 13 years and individual cardiometabolic risk factors at 17 years, using imputed datasets in the ALSPAC cohort (*n* = 1937)

CMR Factors z-score at 17 years (*n* = 1937)	Children's Dietary Inflammatory Score (cDIS), per 1-unit increment					
	cDIS at 7 years		cDIS at 10 years		cDIS at 13 years	
	*β* (95% CI )^a^	*p* value	*β* (95% CI )^a^	*p* value	*β* (95% CI )^a^	*p* value
*Anthropometric*						
Body Mass Index	0.15 (0.05, 0.26)	0.003	0.14 (0.03, 0.24)	0.009	0.07 (− 0.03, 0.17)	0.165
Fat Mass Index^b^	0.05 (0.02, 0.08)	0.001	0.05 (0.02, 0.08)	0.003	0.03 (− 0.001, 0.06)	0.059
Waist circumference	N/A		N/A		N/A	
*Blood lipids*						
Total cholesterol	0.02 (− 0.01,0.05)	0.293	0.00 (− 0.03, 0.03)	0.817	0.01 (− 0.02, 0.04)	0.357
HDL-cholesterol^b^	0.02 (− 0.01, 0.05)	0.183	0.01 (− 0.02, 0.04)	0.387	− 0.01 (− 0.04, 0.02)	0.355
LDL-cholesterol^b^	0.03 (− 0.001, 0.06)	0.055	0.01 (− 0.02, 0.04)	0.492	0.02 (− 0.02, 0.05)	0.321
Triacylglycerol^b^	− 0.01 (− 0.05, 0.02)	0.343	− 0.01 (− 0.04, 0.02)	0.615	− 0.03 (− 0.06, − 0.00)	0.046
*Blood pressure*						
Systolic BP	0.04 (0.01, 0.07)	0.005	0.02 (− 0.01, 0.05)	0.212	0.02 (− 0.01, 0.05)	0.209
Diastolic BP	0.03 (− 0.004, 0.06)	0.095	0.04 (0.01, 0.07)	0.015	0.03 (0.004, 0.06)	0.027
Mean Arterial BP^b^	0.04 (0.01, 0.07)	0.016	0.03 (0.003, 0.06)	0.031	0.03 (0.002, 0.06)	0.039
*Glucose metabolism*						
Insulin	0.03 (− 0.002, 0.06)	0.066	0.05 (0.02, 0.08)	0.003	0.03 (0.001, 0.06)	0.043
Glucose	0.02 (− 0.01, 0.05)	0.209	− 0.00 (− 0.04, 0.03)	0.789	− 0.01 (− 0.04, 0.02)	0.503
HOMA-IR^b^	0.03 (− 0.001, 0.06)	0.055	0.04 (0.01, 0.07)	0.006	0.03 (− 0.002, 0.06)	0.068

*cDIS* energy adjusted children's Dietary Inflammatory Score, *CMR score* Cardiometabolic risk score, *HOMA-IR* Homeostatic Model Assessment of Insulin Resistance, *BP* Blood Pressure, *HDL cholesterol* High-density lipoprotein cholesterol, *LDL cholesterol* low-density lipoprotein cholesterol. ^a^Beta coefficients (95% confidence intervals) derived from multivariable linear regression models adjusted for sex, dietary misreporting, physical activity at 11 and 13 years, mother's highest education level, highest family social class. ^b^Cardiometabolic parameters included in the Cardiometabolic Risk Score

**Table 6:** Association between the children’s Dietary Inflammatory Score (cDIS) at 7, 10 and 13 years and individual cardiometabolic risk factors at 24 years, using imputed datasets in the ALSPAC cohort (*n* = 1957)

CMR Factors z-score at 24 years (*n* = 1957)	Children’s Dietary Inflammatory Score (cDIS), per 1-unit increment					
	cDIS at 7 years		cDIS at 10 years		cDIS at 13 years	
	*β* (95% CI )^a^	*p* value	*β* (95% CI )^a^	*p* value	*β* (95% CI )^a^	*p* value
*Anthropometric*						
Body Mass Index	0.16 (0.09, 0.23)	< 0.001	0.08 (0.01, 0.14)	0.032	0.07 (− 0.00, 0.14)	0.057
Fat Mass Index^b^	0.06 (0.03, 0.09)	< 0.001	0.03 (0.00, 0.06)	0.022	0.03 (0.0002, 0.06)	0.050
Waist circumference	0.05 (0.02, 0.08)	0.002	0.04 (0.01, 0.07)	0.012	0.02 (− 0.01, 0.06)	0.171
*Blood lipids*						
Total cholesterol	0.00 (− 0.03, 0.03)	0.786	0.00 (− 0.02, 0.04)	0.595	0.00 (− 0.03, 0.03)	0.891
HDL-cholesterol^b^	0.03 (− 0.0004, 0.06)	0.053	0.01 (− 0.01, 0.04)	0.335	0.02 (− 0.01, 0.05)	0.267
LDL-cholesterol^b^	0.02 (− 0.01, 0.05)	0.196	0.01 (− 0.02, 0.03)	0.595	0.01 (− 0.12, 0.04)	0.495
Triacylglycerol^b^	0.09 (− 0.02, 0.20)	0.121	0.01 (− 0.02, 0.04)	0.492	− 0.01 (− 0.04, 0.02)	0.742
*Blood pressure*						
Systolic BP	0.05 (0.02, 0.08)	0.003	0.03 (− 0.004, 0.06)	0.085	0.03 (− 0.003, 0.06)	0.074
Diastolic BP	0.04 (0.01, 0.07)	0.004	0.01 (− 0.02, 0.04)	0.508	0.01 (− 0.02, 0.04)	0.347
Mean Arterial BP^b^	0.05 (0.02, 0.08)	0.002	0.02 (− 0.01, 0.05)	0.245	0.02 (− 0.01, 0.05)	0.164
*Glucose metabolism*						
Insulin	0.05 (0.02, 0.08)	0.001	0.03 (− 0.01, 0.06)	0.066	0.03 (− 0.0005, 0.06)	0.054
Glucose	0.00 (− 0.03, 0.03)	0.926	− 0.01 (− 0.04, 0.02)	0.515	0.00 (− 0.03, 0.03)	0.991
HOMA-IR^b^	0.05 (0.02,0.08)	0.001	0.02 (− 0.005, 0.05)	0.103	0.03 (− 0.002, 0.06)	0.069

*cDIS* energy adjusted children's Dietary Inflammatory Score, *CMR score* Cardiometabolic risk score, *HOMA-IR* Homeostatic Model Assessment of Insulin Resistance, *BP* Blood Pressure, *HDL cholesterol* High-density lipoprotein cholesterol, *LDL cholesterol* low-density lipoprotein cholesterol

^a^Beta coefficients (95% confidence intervals) derived from multivariable linear regression models adjusted for sex, dietary misreporting, physical activity at 11 and 13 years, mother's highest education level, highest family social class

^b^Cardiometabolic parameters included in the Cardiometabolic Risk Score

## Discussion

To our knowledge, this is the largest prospective study to assess the associations between the inflammatory potential of the diet in childhood and overall CMR in young adults. Our results show that a higher cDIS at 7 and 10 years was related to an increase in CMR *z*-scores at 17 and 24 years, indicating that a more pro-inflammatory diet during this period of childhood resulted in poorer cardiometabolic health in late adolescence and early adulthood. The associations between the cDIS and CMR scores were largely driven by increased adiposity, blood pressure and insulin resistance. This research supports the concept that inflammation is an important underlying mechanistic pathway connecting diet quality during childhood to the development of CMR factors.

Our results are in line with findings from a Mexican cohort study, which found that cumulative exposure to a pro-inflammatory diet (measured using the C-DII and DII) from infancy to young adulthood was positively associated with a metabolic risk *z*-score in 100 21–22-year-olds (*β* 0.12; *p* = 0.009). The metabolic risk *z*-score included similar components to the CMR score in our study [36]. In contrast, the Project Viva pre-birth USA cohort study did not find any evidence of an association between the DII and a composite metabolic risk score in childhood (the DII was only related to the development of adiposity) [35]. The differences in sample size and age at which the DII score and metabolic risk score were assessed in their study may explain this disparity. Their study calculated a metabolic risk score in 992 children who were 6–10 years old, while the CMR score in our study was calculated in approximately 2000 participants and at 17 and 24 years of age. In ALSPAC we found that variation in values of CMR measurements increased from 17 to 24 years, therefore there may be greater power to detect diet-cardiometabolic associations in adolescents and young adults compared to primary-school aged children. We observed an association between the CMR score (at 17 and 24 years) and cDIS at 7 years but not at 13 years, which could be indicative of a more sensitive period where the effect of dietary induced inflammation during the earlier childhood years had a greater impact on early adulthood CMR factors compared to dietary inflammation during the teenage years. However, this theory would need to be explored and replicated in future studies. It is also worth noting that at 13 years there were higher levels of misreporting of dietary intake (although dietary misreporting was adjusted for in multivariable regression models) and it is likely that parents were less influential in the child’s eating habits than in earlier years, leading to a more changeable diet.

Our findings also align with results from a meta-analysis in adults which concluded that pro-inflammatory diets increased the risk of several CMR factors: MetS, hyperglycaemia and high blood pressure [30]. In addition, they performed a meta-analysis of 6 cohort studies and found the most pro-inflammatory dietary category compared to the most anti-inflammatory category increased risk of cardiometabolic diseases by 35% (HR 1.35, 95% CI 1.13, 1.61) [30]. A longer follow-up of the ALSPAC cohort would be needed before we can examine cardiometabolic disease endpoints.

In terms of the individual CMR factors, inflammatory diet scores in our study were related to several markers of adiposity; participants with more pro-inflammatory diets at 7 and 10 years had higher FMI, BMI and waist circumference *z*-scores at 17 and 24 years. In previous research on the DII and established CMR factors in children, general and/or abdominal obesity (defined using waist-to-hip or waist circumference, BMI z-scores or overweight/obesity cut-offs) was the most consistent factor related to more pro-inflammatory diets [28]. However, the aforementioned meta-analysis in adults did not find an association between the DII score and abdominal obesity [30]. Obesity is recognised for inducing a pro-inflammatory state [3], yet the biological mechanisms by which inflammatory diet can promote obesity are not entirely known. Proposed hypotheses include the influence of pro-inflammatory cytokines on weight gain and appetite stimulation, hypothalamic inflammation from excess of certain nutrients and dietary induced changes in intestinal microbiota [56]. Our findings relating proinflammatory diets to obesity could be relevant for public health preventative strategies, especially since now over a third of children in the UK are overweight or obese when they leave primary school [57]. Furthermore, childhood obesity frequently tracks into adulthood and is a key risk factor for cardiovascular disease [58].

The observed relationship between more pro-inflammatory diets in childhood (7 years and 10 years) and higher blood pressure in early adulthood (17 and 24 years) is in line with findings from a Mexican cohort study, which found that a cumulative proinflammatory diet from infancy to young adulthood was related to higher SBP and DBP in young adults [36]. A cross-sectional study in the USA using NHANES data also reported a positive association between the C-DII and SBP, but only among overweight adolescents [33]. They also found that higher C-DII scores were related to lower DBP. In contrast, the Project Viva cohort study [35] and a Spanish cross-sectional study [59] found no association between the DII and blood pressure (measured in children with a median age of 7.7 years and 12.3 years). Blood pressure was assessed during late adolescence/early adulthood in our study, so age differences may contribute to the discrepancies in results between studies. Similar research using the DII in adults has also produced mixed findings [30, 60]. However, overall, there appears to be evidence of a relationship: individuals in the highest versus lowest DII category had a 13% [OR 1.13 (95% CI 101, 1.27)] increased risk of hypertension in a meta-analysis of 15 observational studies [60]. Plausible mechanisms could be due to the high consumption of fruit and vegetables, common to more anti-inflammatory diets [17, 61], that provide dietary potassium, magnesium, inorganic nitrates and fibre, which have all been shown to lower blood pressure [62]. In addition, markers of chronic inflammation, including CRP, IL-6 and TNR-α have been associated with higher blood pressure [63, 64].

The cDIS in our study was also related to insulin resistance, assessed using the HOMA-IR. This contrasts with the limited previous research in children/adolescents where the DII was not associated with fasting insulin in young children in the Project Viva cohort [35] nor with insulin or fasting glucose in young adults in a Mexican cohort [36]. Our sample size was considerably larger though, which may have increased study power. In addition, research on the DII and glucose metabolism in adults corroborate our findings. A meta-analysis of eight observational studies showed that individuals in the highest versus lowest DII category had a 21% (OR 1.21, 95% CI 1.01, 1.44) higher probability of hyperglycaemia [30]. Another meta-analysis of six studies found that being in the highest compared to the lowest DII category increased HOMA-IR value by 0·19 (*p* = 0·026), although there was substantial heterogeneity between studies [60]. In a cross-sectional study the association between an adapted DII and HOMA-IR was largely explained by scores representing systemic low-grade inflammation, even after adjusting for BMI [65]. Overall, the evidence suggests that chronic low-grade inflammation is one of the pathways by which poor diet quality leads to an impaired action of insulin [66].

The study’s limitations should be considered when interpreting our findings. At birth, the ALSPAC children were relatively representative of the population in the area [39]. However, the 17-year follow-up resulted in some loss to follow-up bias. For instance, the sub-population of the cohort included in our study were more likely to be from a higher social class, have a mother with a higher level of education, and have better cardiometabolic profile in early adulthood. Furthermore, previous research on the index children of ALSPAC has shown that dietary patterns correlate with several socioeconomic factors [67], therefore, children with a less healthy dietary pattern and a lower social class were under-represented in our analysis. This should be considered when generalising our results to the general population. Reporting error and recall bias during dietary data collection is a further limitation. However, diet diaries are generally less prone to misreporting than food frequency questionnaires [68] and we adjusted for validity of dietary reporting in all analyses. We attempted to limit confounding by adjusting for other variables which were identified as confounders of the cDIS-CMR score association: maternal education, family social class and offspring physical activity. Nonetheless, the observational nature of our study means we cannot rule out residual confounding due to measurement error in these data or other unknown confounding factors not included. The construction of the cDIS should also be mentioned [69]. We standardised the individual dietary components of the cDIS using data from our own study population, similar to previous studies [70, 71], since our research aims were to assess the association between inflammatory diets and cardiometabolic health within this cohort and so internal validity was our main priority. Therefore, the cDIS calculations were not designed to be directly compared to the C-DII in other studies which have been standardised using a children’s world database [27].

The strengths of this study include its longitudinal design with 17 years of follow-up of almost 2000 children, starting in childhood. We had repeated measures of both exposure (cDIS) and outcome (CMR score) which meant we were able to evaluate potentially critical/sensitive periods during childhood when the inflammatory potential of the diet might have a greater impact on subsequent cardiometabolic health, as well as cumulative dietary exposure during childhood. In order to minimize attrition bias and increase efficiency and precision of association estimates we imputed missing confounder data [72]. Studying the inflammatory potential of the whole diet, using the cDIS, can be advantageous to studying the inflammatory effect of individual foods or nutrients whose impact on CMR factors may be too small to observe. It is also relevant as nutrients are seldom eaten alone, and so interactions and intercorrelations among nutrients can be taken into account. The nutrients included in the cDIS were energy-adjusted to control for interindividual differences in energy intake. Another strength of our study is the use of a composite CMR score. These scores are increasingly applied in epidemiological research on CMR factors in children [37] because they give a valuable summary of overall cardiometabolic health which can be used as an intermediate pre-clinical outcome in younger populations before overt cardiometabolic diseases are evident. It is also a way to accumulate subtle variation in a range of risk factors included to represent overall risk, since the variation in any individual risk factor may be too subtle to indicate risk on its own. A comprehensive range of individual CMR markers were also studied separately to explore which of the markers in this score were most influenced by dietary-induced inflammation.

In summary, this prospective study showed that consuming a more pro-inflammatory diet during childhood (7–10 years) was associated with a worse overall cardiometabolic profile in late adolescence/early adulthood. This research supports the hypothesis that the inflammatory potential of the diet throughout the life-course, starting from childhood, can increase the risk of developing CMR factors that are predictive of cardiovascular disease and type 2 diabetes. Diet is a major modifiable lifestyle factor which can regulate chronic low-grade inflammation, and inflammation is one of the underlying aetiological mechanisms behind numerous chronic diseases. Therefore, advocating dietary patterns from childhood which are abundant in foods and nutrients with anti-inflammatory properties, while reducing consumption of foods and nutrients that exacerbate inflammation, could be an important strategy to prevent cardiometabolic diseases.

## Supplementary Information

Below is the link to the electronic supplementary material.

Supplementary file1 (DOCX 73 KB)
