# Supplementary material for: The inflammatory potential of the diet in childhood is associated with cardiometabolic risk in adolescence/young adulthood in the ALSPAC birth cohort
Source: Eur J Nutr. 2022 May 20;61(7):3471–86. doi: 10.1007/s00394-022-02860-9 (PMC9464173; doi:10.1007/s00394-022-02860-9)
Supplement: Supplementary file 1 — Supplementary file1 (DOCX 73 KB) [file 394_2022_2860_MOESM1_ESM.docx]

**Supplementary Material: European Journal of Nutrition**

**The inflammatory potential of the diet in childhood is associated with cardiometabolic risk in adolescence/young adulthood in the ALSPAC birth cohort.**

Buckland G^*^, Northstone K, Emmett PM, Taylor CM

**^*^Centre for Academic Child Health, Bristol Medical School, University of Bristol, Bristol, UK**

[**g.buckland@bristol.ac.uk**](mailto:Caroline.m.taylor@bristol.ac.uk)

**Supplementary Table 1:** Comparison of baseline characteristics and cardiometabolic risk factors at 17 years and 24 years in ALSPAC participants who had complete dietary and at all three ages (7, 10 and 13 years) and outcome data at 17 years and/or 24 years compared to those with incomplete data on these variables.

| **Characteristics of the ALSPAC index children** | **Eligible ALSPAC cohort** | | | | | |
| --- | --- | --- | --- | --- | --- | --- |
|  | **N total** | **Incomplete dietary data^a^** | | **Complete dietary data^b^** | | **P-value^d^** |
|  |  | **N** | **% or mean±sd^c^** | **N** | **% or mean±sd** |  |
| ***Baseline Characteristics*** |  |  |  |  |  |  |
| *Sex* |  |  |  |  |  |  |
| Male | 7,592 | 6,309 | 52 | 1,283 | 47 |  |
| Female | 7,277 | 5,820 | 48 | 1,457 | 53 | <0.001 |
| BMI at 10 years, kg/m^2^ | 7,455 | 4,734 | 18.4 ± 3.3 | 2,721 | 18.1 ± 2.9 | 0.004 |
| Energy intake at 7 years, kJ/d | 7,264 | 4,524 | 7,117.3 ± 1,346.9 | 2,740 | 7,233.8 ±1,270.5 | <0.001 |
| Energy intake at 10 years, kJ/d | 7,451 | 4,711 | 7,743.0 ± 1,665.8 | 2,740 | 7,858.7 ±1,538.9 | <0.001 |
| Energy intake at 13 years, kJ/d | 6,096 | 3,356 | 8,174.8 ± 2,271.8 | 2,740 | 8,244.9 ±2,081.7 | 0.025 |
| Maternal age at delivery, years | 13,957 | 11,325 | 27.6 ± 5.0 | 2,632 | 29.5 ± 4.4 | <0.001 |
| Maternal pre-pregnancy BMI, kg/m^2^ | 11,516 | 9,066 | 23.0 ± 3.9 | 2,450 | 22.7 ± 3.6 | 0.004 |
| Maternal highest education |  |  |  |  |  |  |
| CSE, Vocational or O level | 8,016 | 6,774 | 69.1 | 1,242 | 47.9 |  |
| A-level or Degree | 4,382 | 3,029 | 30.9 | 1,353 | 52.1 | <0.001 |
| Highest household social class |  |  |  |  |  |  |
| I and II | 2,982 | 2,104 | 23.6 | 878 | 34.7 |  |
| III, IV and V | 8,472 | 6,818 | 76.4 | 1,654 | 65.3 | <0.001 |
| ***CMR factors at 17 years*** |  |  |  |  |  |  |
| Fat Mass Index, kg/m^2^ | 4,824 | 2,378 | 6.7 ± 4.0 | 2,446 | 6.0 ± 3.5 | <0.001 |
| HDL-cholesterol, mmol/L | 3,284 | 1,206 | 1.3 ± 0.3 | 2,078 | 1.3 ±0.3 | 0.018 |
| LDL-cholesterol, mmol/L | 3,284 | 1,206 | 2.1 ± 0.6 | 2,078 | 2.1 ± 0.6 | 0.503 |
| Triacylglycerol, mmol/L | 3,284 | 1,206 | 0.9 ± 0.4 | 2,078 | 0.8 ± 0.3 | 0.002 |
| MAP, mmHg | 4,656 | 2,234 | 82.0 ± 6.4 | 2,422 | 81.3 ± 5.9 | <0.001 |
| HOMA-IR | 3,214 | 1,157 | 2.3 ± 3.4 | 2,057 | 1.7 ± 1.2 | <0.001 |
| ***CMR factors at 24 years*** |  |  |  |  |  |  |
| Fat Mass Index, kg/m^2^ | 3,859 | 1,759 | 8.9 ± 19.1 | 2,100 | 7.8 ± 3.7 | <0.001 |
| HDL-cholesterol, mmol/L | 3,252 | 1,232 | 1.5 ± 0.4 | 2,020 | 1.6 ± 0.4 | 0.334 |
| LDL-cholesterol, mmol/L | 3,250 | 1,230 | 2.5 ± 0.8 | 2,020 | 2.4 ± 0.7 | 0.694 |
| Triacylglycerol, mmol/L | 3,251 | 1,231 | 1.0 ± 0.7 | 2,020 | 0.9 ± 0.5 | 0.087 |
| MAP, mmHg | 3,993 | 1,851 | 83.3 ± 8.4 | 2,142 | 83.3 ± 8.0 | 0.939 |
| HOMA-IR | 3,252 | 1,232 | 2.6 ± 3.3 | 2,020 | 2.2 ± 2.2 | 0.030 |
| Abbreviations; CMR: Cardiometabolic Risk. HDL: High-density lipoprotein cholesterol. LDL: Low-density lipoprotein cholesterol. MAP: Mean arterial blood pressure. HOMA-IR: Homeostatic Model Assessment of Insulin Resistance. | | | | | | |
| ^a^Incomplete dietary data refers to missing dietary data at ≥1 age (7, 10, 13 years) and missing CMR score at 17 or 24 years. ^b^Complete dietary data refers to dietary data collected at all three ages (7, 10 and 13 years) and complete CMR score at 17 or 24 years. ^c^Percentage (all such values and refers to column percentages) or Mean ± SD (all such values). ^d^Chi-squared test for categorical variables and Kruskal-Wallis test for continuous variables. | | | | | | |

**Supplementary Table 2.** Comparison of characteristics of ALSPAC participants with imputed and observed data.

| **Characteristics of the ALSPAC index children** | **Dataset with outcome at 17 years (n=1,937)** | | | **Dataset with outcome at 24 years (n=1,957)** | | |
| --- | --- | --- | --- | --- | --- | --- |
|  | **Imputed** | **Observed** | **% of data imputed** | **Imputed** | **Observed** | **% of data imputed** |
|  | **Mean ± SE or %** | |  | **Mean ± SE or %** | |  |
| **Maternal highest education** |  |  |  |  |  |  |
| CSE or Vocational training | 14.2 | 14.2 |  | 12.3 | 12.2 |  |
| O-level | 32.1 | 32.2 |  | 33.5 | 33.6 |  |
| A-level | 31.0 | 31.0 |  | 30.6 | 30.6 |  |
| Degree or higher | 22.7 | 22.7 | 5.1 | 23.6 | 23.6 | 5.1 |
| **Highest household social class** |  |  |  |  |  |  |
| I | 5.1 | 5.1 |  | 5.9 | 5.8 |  |
| II | 30.4 | 30.4 |  | 30.5 | 30.6 |  |
| III non-manual | 28.6 | 28.7 |  | 28.6 | 28.6 |  |
| III manual | 22.4 | 22.5 |  | 22.9 | 22.8 |  |
| IV and V | 13.4 | 13.3 | 7.5 | 12.1 | 12.1 | 7.3 |
| **Accuracy of dietary reporting - 7 years** |  |  |  |  |  |  |
| Under-reporting | 9.3 | 9.3 |  | 10.0 | 10.1 |  |
| Accurate reporting | 75.5 | 75.6 |  | 76.3 | 76.4 |  |
| Over-reporting | 15.2 | 15.0 | 0.5 | 13.8 | 13.7 | 0.4 |
| **Accuracy of dietary reporting - 10 years** |  |  |  |  |  |  |
| Under-reporting | 30.1 | 30.2 |  | 32.6 | 32.6 |  |
| Accurate reporting | 67.1 | 67.2 |  | 64.5 | 64.6 |  |
| Over-reporting | 2.7 | 2.7 | 0.4 | 2.9 | 2.9 | 0.3 |
| **Accuracy of dietary reporting - 13 years** |  |  |  |  |  |  |
| Under-reporting | 60.1 | 60.1 |  | 60.2 | 60.2 |  |
| Accurate and over-reporting | 39.9 | 39.9 | 0.6 | 39.8 | 39.8 | 0.5 |
| **Moderate-to-vigorous physical activity at 11 years** |  |  |  |  |  |  |
| <20 mins | 49.1 | 49.1 |  | 51.9 | 52 |  |
| ≥20 to <40 mins | 36.9 | 36.9 |  | 35.4 | 35.3 |  |
| ≥40mins | 14.0 | 14.0 | 13.5 | 12.8 | 12.7 | 14.8 |
| **Moderate-to-vigorous physical activity at 13 years** |  |  |  |  |  |  |
| <20 mins | 48.0 | 47.8 |  | 50.1 | 50.5 |  |
| ≥20 to <40 mins | 36.2 | 36.2 |  | 35.2 | 35.2 |  |
| ≥40mins | 16.2 | 16.0 | 21.2 | 14.7 | 14.3 | 21.3 |

**Supplementary Table 3**. Nutrient intakes and corresponding inflammatory weights for the 24 items included in the children’s Dietary Inflammatory Score (cDIS), and median intake (25-75^th^ percentiles) of cDIS at 7, 10 and 13 years in the ALSPAC cohort (n=2,740)

| **Nutrient** | **Inflammatory weight** | **Daily intake at 7 years** | | **Daily intake at 10 years** | | **Daily intake at 13 years** | |
| --- | --- | --- | --- | --- | --- | --- | --- |
|  |  | **Median** | **IQR** | **Median** | **IQR** | **Median** | **IQR** |
| Energy (kJ) | 0.180 | 1,702 | (1,511-1,902) | 1,849 | (1,623-2,101) | 1,918 | (1617-2270) |
| Macronutrients |  |  |  |  |  |  |  |
| Carbohydrate (g) | 0.097 | 229.2 | (202.6-257.9) | 247.3 | (214.7-284.5) | 256.5 | (212.9-305.2) |
| Protein (g) | 0.021 | 54.9 | (47.5-63.5) | 61.3 | (51.7-71.8) | 67.9 | (55.6-81.1) |
| Fibre (g) | -0.663 | 10.3 | (8.4-12.6) | 11.3 | (9.1-13.8) | 12.5 | (9.8-15.7) |
| Fats |  |  |  |  |  |  |  |
| Total fat (g) | 0.298 | 68.3 | (58.6-78.9) | 74.0 | (62.0-86.6) | 74.8 | (60.9-91.9) |
| Saturated fat (g) | 0.373 | 27.3 | (22.5-32.8) | 28.1 | (23.0-34.3) | 27.7 | (21.7-35.3) |
| MUFA (g) | -0.009 | 22.8 | (19.4-26.6) | 25.1 | (21.0-29.6) | 25.2 | (19.9-31.2) |
| PUFA (g) | -0.337 | 10.3 | (8.2-12.9) | 11.6 | (9.2-14.8) | 12.2 | (9.1-15.7) |
| Cholesterol (mg) | 0.110 | 157.5 | (120.5-208.5) | 170.2 | (126.6-230.1) | 178.4 | (129.3-245.8) |
| Vitamins |  |  |  |  |  |  |  |
| Vitamin A (RE) (μg) | -0.401 | 613.1 | (451.3-829.1) | 644.5 | (447.8-898.3) | 653.3 | (439.8-931.6) |
| Vitamin B6 (mg) | -0.365 | 1.7 | (1.4-2.1) | 1.8 | (1.5-2.2) | 1.9 | (1.5-2.4) |
| Vitamin B12 (μg) | 0.106 | 3.5 | (2.6-4.6) | 3.3 | (2.4-4.3) | 4.0 | (2.7-5.6) |
| Vitamin C (mg) | -0.424 | 69.7 | (42.4-109.0) | 78.4 | (44.5-123.0) | 93.6 | (52.0-145.0) |
| Vitamin D (μg) | -0.446 | 2.3 | (1.7-3.0) | 2.5 | (1.8-3.3) | 2.4 | (1.6-3.4) |
| Vitamin E (mg) | -0.419 | 7.5 | (5.9-9.8) | 8.3 | (6.5-11.0) | 8.4 | (6.3-11.2) |
| Folic acid (μg) | -0.190 | 193.6 | (156.1-237.7) | 205.6 | (161.6-253.9) | 224.7 | (174.1-288.1) |
| β-Carotene (μg) | -0.584 | 524.5 | (270.4-845.8) | 568.3 | (264.8-990.6) | 601.8 | (286.9-1,095.1) |
| Thiamin (mg) | -0.098 | 1.3 | (1.1-1.7) | 1.4 | (1.1-1.7) | 1.5 | (1.2-1.9) |
| Riboflavin (mg) | -0.068 | 5.9 | (5.0-7.1) | 1.6 | (1.2-2.0) | 1.7 | (1.2-2.2) |
| Niacin (mg) | -0.246 | 14.7 | (12.2-17.8) | 16.3 | (13.3-20.1) | 18.2 | (14.1-22.9) |
| Minerals |  |  |  |  |  |  |  |
| Selenium (μg) | -0.191 | 51.7 | (41.2-63.2) | 57.3 | (45.4-70.0) | 60.8 | (46.3-77.1) |
| Iron (mg) | 0.032 | 8.2 | (7.0-9.6) | 8.9 | (7.6-10.5) | 9.8 | (8.0-12.0) |
| Magnesium (mg) | -0.484 | 197.9 | (168.6-230.4) | 214.1 | (181.3-250.8) | 234.3 | (191.3-280.4) |
| Zinc (mg) | -0.313 | 5.9 | (5.0-7.1) | 6.6 | (5.5-7.9) | 7.3 | (5.9-8.9) |

**Supplementary Table 4.** Multivariable linear regression models for the relationship between the children’s Dietary Inflammatory Score (cDIS) at 7, 10 and 13 years and cardiometabolic risk score at 17 years in the ALSPAC cohort for complete-case analysis (n=1,258).

| **children's Dietary Inflammatory Score (cDIS)^a^** | **Cardiometabolic Risk (CMR) score at 17 years** | | | | | | |
| --- | --- | --- | --- | --- | --- | --- | --- |
|  |  | **Crude** | | **Minimally Adjusted^b^** | | **Fully Adjusted^c^** | |
|  | **N** | **ß (95%CI)** | **P-value** | **ß (95%CI)** | **P-value** | **ß (95%CI)** | **P-value** |
| ***cDIS at 7 years*** |  | | | | | | |
| Tertile 1 | 423 | Reference | | Reference | | Reference | |
| Tertile 2 | 419 | -0.03 (-0.22, 0.17) | 0.781 | 0.00 (-0.20,0.19) | 0.975 | -0.02 (-0.21,0.18) | 0.869 |
| Tertile 3 | 416 | 0.24 (0.05,0.44) | 0.016 | 0.30 (0.10,0.50) | 0.003 | 0.28 (0.08,0.48) | 0.007 |
| Continuous^d^ | 1,258 | 0.07 (0.02,0.12) | 0.010 | 0.09 (0.04,0.14) | 0.001 | 0.08 (0.03,0.14) | 0.004 |
| ***cDIS at 10 years*** |  | | | | | | |
| Tertile 1 | 402 | Reference | | Reference | | Reference | |
| Tertile 2 | 428 | 0.01 (-0.19,0.21) | 0.907 | 0.04 (-0.16,0.23) | 0.717 | 0.02 (-0.17,0.22) | 0.810 |
| Tertile 3 | 428 | 0.11 (-0.09,0.31) | 0.271 | 0.18 (-0.02,0.38) | 0.071 | 0.13 (-0.07,0.33) | 0.192 |
| Continuous^d^ | 1,258 | 0.05 (-0.00,0.10) | 0.074 | 0.07 (0.02,0.13) | 0.011 | 0.06 (0.00,0.11) | 0.041 |
| ***cDIS at 13 years*** |  | | | | | | |
| Tertile 1 | 395 | Reference | | Reference | | Reference | |
| Tertile 2 | 437 | -0.09 (-0.29,0.11) | 0.361 | -0.01 (-0.21,0.19) | 0.911 | -0.03 (-0.23,0.17) | 0.767 |
| Tertile 3 | 426 | 0.03 (-0.16,0.23) | 0.733 | 0.17 (-0.04,0.37) | 0.111 | 0.13 (-0.07,0.34) | 0.205 |
| Continuous^d^ | 1,258 | 0.00 (-0.05,0.05) | 0.879 | 0.03 (-0.02,0.09) | 0.193 | 0.03 (-0.03,0.08) | 0.355 |
| ***cDIS tracking 7-10-13 years***^e^ |  | | | | | | |
| cDIS low | 367 | Reference | | Reference | | Reference | |
| cDIS mixed | 190 | -0.04 (-0.30,0.21) | 0.746 | -0.02 (-0.27,0.24) | 0.900 | -0.04 (-0.29,0.21) | 0.757 |
| cDIS medium | 340 | -0.12 (-0.33,0.10) | 0.283 | -0.07 (-0.28,0.15) | 0.540 | -0.08 (-0.29,0.13) | 0.458 |
| cDIS high | 361 | 0.16 (-0.05,0.37) | 0.134 | 0.28 (0.06,0.49) | 0.010 | 0.23 (0.02,0.44) | 0.034 |
| ^a^cDIS: energy adjusted children's Dietary Inflammatory score. ^b^Minimally Adjusted: Multivariable regression model adjusted for sex and dietary misreporting. ^c^Fully Adjusted: Multivariable regression model adjusted for sex, dietary misreporting, maternal highest education level, family highest social class and physical activity level at 11 years (for analysis of cDIS at 7 and 10 years) and physical activity at 13 years (for analysis of cDIS at 13 years). ^d^Estimated mean change in CMR z-score associated with a 1 unit increase in E-cDIS. ^e^cDIS tracking; low indicates first tertile of cIDS at least twice from 7-10-13 years, mixed indicates different tertiles of cDIS at 7-10-13 years, medium indicates second tertile of cDIS at least twice from 7-10-13 years, high indicates high tertile of cDIS at least twice from 7-10-13 years. | | | | | | | |

**Supplementary Table 5.** Multivariable linear regression models for the relationship between the children’s Dietary Inflammatory Score (cDIS) at 7, 10 and 13 years and cardiometabolic risk score at 24 years, in the ALSPAC cohort for complete-case analysis (n=1,266).

| **children's Dietary Inflammatory Score (cDIS)^a^** | **Cardiometabolic Risk (CMR) score at 24 years** | | | | | | |
| --- | --- | --- | --- | --- | --- | --- | --- |
|  |  | **Crude** | | **Minimally Adjusted^b^** | | **Fully Adjusted^c^** | |
|  | **N** | **ß (95%CI)** | **P-value** | **ß (95%CI)** | **P-value** | **ß (95%CI)** | **P-value** |
| ***cDIS at 7 years*** |  | | | | | | |
| Tertile 1 | 432 | Reference |  | Reference |  | Reference |  |
| Tertile 2 | 426 | 0.23 (0.03,0.44) | 0.027 | 0.26 (0.05,0.47) | 0.014 | 0.25 (0.05,0.46) | 0.017 |
| Tertile 3 | 408 | 0.31 (0.10,0.52) | 0.003 | 0.37 (0.16,0.59) | 0.001 | 0.34 (0.12,0.55) | 0.002 |
| Continuous^d^ | 1,266 | 0.09 (0.03,0.15) | 0.002 | 0.11 (0.06,0.17) | <0.001 | 0.10 (0.04,0.16) | 0.001 |
| ***cDIS at 10 years*** |  | | | | | | |
| Tertile 1 | 413 | Reference |  | Reference |  | Reference |  |
| Tertile 2 | 433 | 0.15 (-0.06,0.36) | 0.172 | 0.17 (-0.04,0.38) | 0.110 | 0.15 (-0.06,0.36) | 0.165 |
| Tertile 3 | 420 | 0.11 (-0.10,0.32) | 0.313 | 0.16 (-0.05,0.38) | 0.131 | 0.12 (-0.09,0.33) | 0.262 |
| Continuous^d^ | 1,266 | 0.05 (-0.00,0.11) | 0.067 | 0.07 (0.01,0.13) | 0.015 | 0.06 (0.00,0.12) | 0.042 |
| ***cDIS at 13 years*** |  | | | | | | |
| Tertile 1 | 418 | Reference |  | Reference |  | Reference |  |
| Tertile 2 | 432 | 0.08 (-0.13,0.29) | 0.463 | 0.13 (-0.08,0.34) | 0.221 | 0.10 (-0.11,0.31) | 0.328 |
| Tertile 3 | 416 | 0.07 (-0.14,0.28) | 0.495 | 0.18 (-0.03,0.40) | 0.098 | 0.14 (-0.08,0.35) | 0.222 |
| Continuous^d^ | 1,266 | 0.03 (-0.03,0.08) | 0.359 | 0.06 (0.00,0.12) | 0.035 | 0.05 (-0.01,0.10) | 0.118 |
| ***cDIS tracking 7-10-13 years^e^*** |  | | | | | | |
| cDIS low | 384 | Reference |  | Reference |  | Reference |  |
| cDIS mixed | 184 | 0.40 (0.13,0.67) | 0.004 | 0.40 (0.13,0.67) | 0.004 | 0.35 (0.08,0.62) | 0.012 |
| cDIS medium | 344 | 0.25 (0.03,0.48) | 0.028 | 0.29 (0.06,0.51) | 0.013 | 0.27 (0.04,0.49) | 0.019 |
| cDIS high | 354 | 0.23 (0.01,0.45) | 0.043 | 0.31 (0.09,0.54) | 0.006 | 0.26 (0.04,0.49) | 0.022 |
| ^a^cDIS: energy-adjusted children's Dietary Inflammatory score. ^b^Minimally Adjusted: Multivariable regression model adjusted for sex and dietary misreporting. ^c^Fully Adjusted: Multivariable regression model adjusted for sex, dietary misreporting, maternal highest education level, family highest social class and physical activity level at 11 years (for analysis of cDIS at 7 and 10 years) and physical activity at 13 years (for analysis of cDIS at 13 years). ^d^Estimated mean change in CMR z-score associated with a 1 unit increase in cDIS. ^e^cDIS tracking: low indicates first tertile of cIDS at least twice from 7-10-13 years, mixed indicates different tertiles of cDIS at 7-10-13 years, medium indicates second tertile of cDIS at least twice from 7-10-13 years, high indicates high tertile of cDIS at least twice from 7-10-13 years. | | | | | | | |

**Supplementary Table 6.** Sensitivity analysis for the association between the children’s Dietary Inflammatory Score (cDIS) at 7, 10 and 13 years and CMR score at 17 and 24 years, using complete-case datasets in the ALSPAC cohort.

| **Children's Dietary Inflammatory Score (cDIS)^a^** | **Cardiometabolic Risk (CMR) score at 17 years** | | | **Cardiometabolic Risk (CMR) score at 24 years** | | |
| --- | --- | --- | --- | --- | --- | --- |
|  | **N** | **ß (95%CI)^a^** | **P-value** | **N** | **ß (95%CI)^a^** | **P-value** |
| ***cDIS (continuous) at 7 years*** |  | | |  | | |
| Full analysis (complete case) | 1,258 | 0.08 (0.03,0.14) | 0.004 | 1,266 | 0.10 (0.04,0.16) | 0.001 |
| Excluding ppt without 3 days of DD | 1,112 | 0.07 (0.02,0.13) | 0.014 | 1,116 | 0.09 (0.03,0.15) | 0.005 |
| Additional adjustment for BMI^b^ | 1,258 | 0.05 (0.00,0.11) | 0.044 | 1,266 | 0.08 (0.02,0.14) | 0.006 |
| ***cDIS (continuous) at 10 years*** |  | | |  | | |
| Full analysis (complete case) | 1,258 | 0.06 (0.00,0.11) | 0.041 | 1,266 | 0.06 (0.00,0.12) | 0.042 |
| Excluding ppt without 3 days of DD | 1,076 | 0.06 (-0.00,0.12) | 0.058 | 1,084 | 0.06 (-0.00,0.12) | 0.068 |
| Additional adjustment for BMI^b^ | 1,251 | 0.03 (-0.02,0.08) | 0.284 | 1,262 | 0.04 (-0.02,0.09) | 0.206 |
| ***cDIS (continuous) at 13 years*** |  | | |  | | |
| Full analysis (complete case) | 1,258 | 0.03 (-0.03,0.08) | 0.355 | 1,266 | 0.05 (-0.01,0.10) | 0.118 |
| Excluding ppt without 3 days of DD | 1,027 | 0.05 (-0.01,0.11) | 0.096 | 1,041 | 0.06 (-0.01,0.12) | 0.085 |
| Additional adjustment for BMI^b^ | 1,258 | 0.001 (-0.05,0.05) | 0.976 | 1,264 | 0.03 (-0.02,0.08) | 0.265 |
| Abbreviations: DD- Diet Diaries. ppt- participants ^a^Multivariable regression model adjusted for sex, dietary misreporting, maternal highest education level, family highest social class and physical activity level at 11 years (for analysis of cDIS at 7 and 10 years) and physical activity at 13 years (for analysis of cDIS at 13 years). Beta-coefficients represent estimated mean change in CMR z-score associated with a 1 unit increase in cDIS. ^b^Additional adjustment for BMI refers to BMI at age of dietary data collection | | | | | | |

**Supplementary Table 7.** Association between the children’s Dietary Inflammatory Score (cDIS) at 7, 10 and 13 years and individual cardiometabolic risk factors at 17 years, using complete-case datasets in the ALSPAC cohort (n=1,258).

| **CMR factor z-score at 17 years (n=1,258)** | **children's Dietary Inflammatory Score (cDIS), per 1-unit increment** | | | | | |  |
| --- | --- | --- | --- | --- | --- | --- | --- |
|  | **cDIS at 7 years** | | **cDIS at 10 years** | | **cDIS at 13 years** | |  |
|  | **ß (95%CI)^a^** | **P-value** | **ß (95%CI)^a^** | **P-value** | **ß (95%CI)^a^** | **P-value** |  |
| ***Anthropometric*** |  | | | | | |  |
| Body Mass Index | 0.19 (0.06,0.32) | 0.004 | 0.14 (0.01,0.26) | 0.031 | 0.12 (-0.00,0.24) | 0.058 |  |
| Fat Mass Index^b^ | 0.05 (0.02,0.09) | 0.005 | 0.05 (0.01,0.08) | 0.014 | 0.03 (-0.00,0.07) | 0.053 |  |
| ***Blood lipids*** |  |  |  |  |  |  |  |
| Total cholesterol | 0.02 (-0.02,0.06) | 0.292 | -0.01 (-0.05,0.03) | 0.664 | 0.01 (-0.03,0.05) | 0.656 |  |
| HDL-cholesterol^b^ | 0.03 (-0.01,0.06) | 0.156 | 0.03 (-0.00,0.07) | 0.086 | -0.001 (-0.04,0.03) | 0.890 |  |
| LDL-cholesterol^b^ | 0.04 (-0.00,0.07) | 0.067 | 0.01 (-0.03,0.05) | 0.612 | 0.02 (-0.02,0.06) | 0.348 |  |
| Triacylglycerol^b^ | -0.001 (-0.04,0.03) | 0.808 | -0.02 (-0.06,0.01) | 0.212 | -0.03 (-0.06,0.01) | 0.150 |  |
| ***Blood pressure*** |  |  |  |  |  |  |  |
| Systolic BP | 0.06 (0.02,0.10) | 0.003 | 0.02 (-0.02,0.06) | 0.281 | 0.01 (-0.03,0.05) | 0.621 |  |
| Diastolic BP | 0.04 (0.00,0.08) | 0.049 | 0.04 (-0.00,0.08) | 0.052 | 0.03 (-0.01,0.06) | 0.177 |  |
| Mean Arterial BP^b^ | 0.05 (0.01,0.09) | 0.007 | 0.03 (-0.00,0.07) | 0.077 | 0.02 (-0.02,0.06) | 0.249 |  |
| ***Glucose metabolism*** |  |  |  |  |  |  |  |
| Insulin | 0.03 (-0.00,0.07) | 0.076 | 0.05 (0.01,0.08) | 0.021 | 0.02 (-0.02,0.06) | 0.294 |  |
| Glucose | 0.02 (-0.01,0.06) | 0.203 | -0.01 (-0.05,0.03) | 0.601 | -0.02 (-0.05,0.02) | 0.408 |  |
| HOMA-IR^b^ | 0.04 (-0.00,0.07) | 0.063 | 0.04 (0.00,0.08) | 0.033 | 0.02 (-0.02,0.05) | 0.375 |  |
| Abbreviations: cDIS: energy adjusted children's Dietary Inflammatory Score; CMR score: Cardiometabolic risk score. HOMA-IR: Homeostatic Model Assessment of Insulin Resistance. BP: Blood Pressure. HDL-cholesterol: High-density lipoprotein cholesterol. LDL-cholesterol: low-density lipoprotein cholesterol. ^a^Beta coefficients (95% confidence intervals) derived from multivariable linear regression models adjusted for sex, dietary misreporting, physical activity at 11 and 13 years, mother's highest education level, highest family social class. ^b^Cardiometabolic parameters included in the Cardiometabolic Risk Score. | | | | | | | |

**Supplementary Table 8.** Association between the children’s Dietary Inflammatory Score (cDIS) at 7, 10 and 13 years and individual cardiometabolic risk factors at 24 years, using complete-case datasets in the ALSPAC cohort (n=1,266).

| **CMR factor z-score at 17 years (n=1,266)** | **children's Dietary Inflammatory Score (cDIS), per 1-unit increment** | | | | | |
| --- | --- | --- | --- | --- | --- | --- |
|  | **cDIS at 7 years** | | **cDIS at 10 years** | | **cDIS at 13 years** | |
|  | **ß (95%CI)^a^** | **P-value** | **ß (95%CI)^a^** | **P-value** | **ß (95%CI)^a^** | **P-value** |
| ***Anthropometric*** |  | | | | | |
| Body Mass Index | 0.21 (0.12,0.29) | <0.001 | 0.10 (0.01,0.19) | 0.022 | 0.09 (0.01,0.18) | 0.035 |
| Fat Mass Index^b^ | 0.08 (0.05,0.12) | <0.001 | 0.04 (0.01,0.08) | 0.022 | 0.04 (0.00,0.07) | 0.034 |
| Waist circumference | 0.07 (0.03,0.11) | <0.001 | 0.04 (-0.00,0.08) | 0.061 | 0.04 (-0.00,0.08) | 0.083 |
| ***Blood lipids*** |  |  |  |  |  |  |
| Total cholesterol | 0.01 (-0.03,0.04) | 0.756 | 0.01 (-0.03,0.04) | 0.757 | -0.00 (-0.04,0.03) | 0.813 |
| HDL-cholesterol^b^ | 0.04 (-0.00,0.07) | 0.051 | 0.03 (-0.01,0.06) | 0.153 | 0.02 (-0.02,0.06) | 0.272 |
| LDL-cholesterol^b^ | 0.02 (-0.02,0.06) | 0.285 | 0.02 (-0.02,0.06) | 0.291 | 0.01 (-0.03,0.04) | 0.753 |
| Triacylglycerol^b^ | 0.01 (-0.03,0.04) | 0.781 | 0.01 (-0.03,0.05) | 0.557 | -0.01 (-0.05,0.03) | 0.539 |
| ***Blood pressure*** |  |  |  |  |  |  |
| Systolic BP | 0.04 (-0.00,0.07) | 0.067 | 0.02 (-0.01,0.06) | 0.210 | 0.02 (-0.01,0.06) | 0.191 |
| Diastolic BP | 0.04 (-0.00,0.07) | 0.064 | 0.01 (-0.03,0.04) | 0.735 | 0.02 (-0.02,0.05) | 0.368 |
| Mean Arterial BP^b^ | 0.04 (0.00,0.08) | 0.043 | 0.01 (-0.02,0.05) | 0.452 | 0.02 (-0.02,0.06) | 0.245 |
| ***Glucose metabolism*** |  |  |  |  |  |  |
| Insulin | 0.07 (0.03,0.10) | <0.001 | 0.04 (-0.00,0.07) | 0.052 | 0.03 (-0.00,0.07) | 0.061 |
| Glucose | -0.01 (-0.05,0.03) | 0.612 | -0.02 (-0.06,0.01) | 0.236 | 0.02 (-0.02,0.05) | 0.330 |
| HOMA-IR^b^ | 0.06 (0.02,0.10) | 0.001 | 0.03 (-0.01,0.07) | 0.096 | 0.04 (-0.00,0.07) | 0.057 |
| Abbreviations: cDIS: energy-adjusted children's Dietary Inflammatory Score; CMR score: Cardiometabolic risk score. HOMA-IR: Homeostatic Model Assessment of Insulin Resistance. BP: Blood Pressure. HDL-cholesterol: High-density lipoprotein cholesterol. LDL-cholesterol: low-density lipoprotein cholesterol. ^a^Beta coefficients (95% confidence intervals) derived from multivariable linear regression models adjusted for sex, dietary misreporting, physical activity at 11 and 13 years, mother's highest education level, highest family social class. ^b^Cardiometabolic parameters included in the Cardiometabolic Risk Score. | | | | | | |
